# The Society of Women Journalists

**Published:** 1917-09-01

**Authors:** Mary MacLeod Moore

**Affiliations:** 10 St. Bride's Avenue, Fleet Street, London, E.C. 4


### THE EDITOR'S LETTER-BOX.

### The Society of Women Journalists.

*To the Editor of* The Hospital.

Sir,—I see in the *Sunday Times* an article from The Hospital in which I am mentioned as chairman of the Canadian Society of Women Journalists. Will you be so good as to correct this statement in your paper, as I am not officially connected with any Canadian society whatever?—Believe me, yours truly,

Mary MacLeod Moore.

10 St. Bride's Avenue, Fleet Street, London, E.C. 4, August 27, 1917.

[We have pleasure in publishing this disclaimer from Miss Mary MacLeod Moore, chairman of the Society of Women Journalists. Is she not a Canadian journalist too?—Ed. The Hospital.]

